# Galvanic Manufacturing in the Cities of Russia: Potential Source of Ambient Nanoparticles

**DOI:** 10.1371/journal.pone.0110573

**Published:** 2014-10-20

**Authors:** Kirill S. Golokhvast, Anna A. Shvedova

**Affiliations:** 1 Scientific Educational Center of Nanotechnology, Far Eastern Federal University, Vladivostok, Russian Federation; 2 Pathology and Physiology Research Branch/NIOSH/CDC, Morgantown, West Virginia, United States of America; 3 Department Physiology and Pharmacology, School of Medicine, West Virginia University, Morgantown, West Virginia, United States of America; RMIT University, Australia

## Abstract

Galvanic manufacturing is widely employed and can be found in nearly every average city in Russia. The release and accumulation of different metals (Me), depending on the technology used can be found in the vicinities of galvanic plants. Under the environmental protection act in Russia, the regulations for galvanic manufacturing do not include the regulations and safety standards for ambient ultrafine and nanosized particulate matter (PM). To assess whether Me nanoparticles (NP) are among environmental pollutants caused by galvanic manufacturing, the level of Me NP were tested in urban snow samples collected around galvanic enterprises in two cities. Employing transmission electronic microscopy, energy-dispersive X-ray spectroscopy, and a laser diffraction particle size analyzer, we found that the size distribution of tested Me NP was within 10–120 nm range. This is the first study to report that Me NP of Fe, Cr, Pb, Al, Ni, Cu, and Zn were detected around galvanic shop settings.

## Introduction

Galvanics is a technology involving electrolytic precipitation of a thin layer of metal (Me) on a surface of pure Me or alloys. The products of galvanics can be used for protection from corrosion, to increase wear resistance, to protect from cementation, and for decorative purposes [1].

Many industries, including automotive, electronics, aerospace, hardware, jewelry, heavy equipment, appliances, tires, and telecommunications, use Me finishing for manufacturing goods [2]. The electroplating, plating, polishing, anodizing, and coloring industry is classified under the Standard Industrial Classification (SIC) code 3471 and includes establishments primarily engaged in all types of Me finishing [3]. Metallic coatings change the surface properties of the workpiece forming a composite material with new properties that could not be achieved by the material alone. The coating’s function is usually as a durable, corrosion-resistant protective layer, while the core material provides a load-bearing function [4]. Common coating materials used include aluminum, lead, tin, zinc, and combinations of these metals.

It has been previously shown that airborne particulate matter (PM) collected from various urban and industrial sites contained a number of hard and soluble Me of Cd, Cu, Mn, Ni, Pb, Zn [5]. The potential adverse effects of hard and soluble metals on the environment and on habitat species are of major concern [6], [7]. Studies conducted at a waste water treatment plant, which is in close proximity to a Ni/Cr plating plant, showed accumulation of different hard Me including Cu, Ni, Cr and Zn [8], [9].

Recent epidemiological studies have shown a strong association between daily morbidity and mortality with increased air pollution [10]–[14]. The hazard posed by galvanic productions to an urban environment is an emerging issue that recently drew public attention and concern [15]–[18]. Chemical analysis of washing and sewage effluents coming to adjacent aquatic sources from locally producing small shops revealed the release of a number of harmful impurities including a high level of heavy Me that exceeded the environmental standard in Russia [19]. A high source of residual hazardous Me (Fe, Cr, Zn, Cu, Pb, Cd, Sn, Ni) and an elevated level of strong acids (e.g., hydrochloric, sulfuric, nitric, phosphoric, hydrocyanic and fluoric), used for electro-plating and/or galvanic fabrications, were seen in water outside of producing areas [19].

No published data is currently available regarding Me NP found around galvanic shops. Therefore, the goal of the current study was to assess whether Me NP could be traceable in snow sediments near industrial galvanic plants.

## Methods

### Sample collections

**Figure 1:**
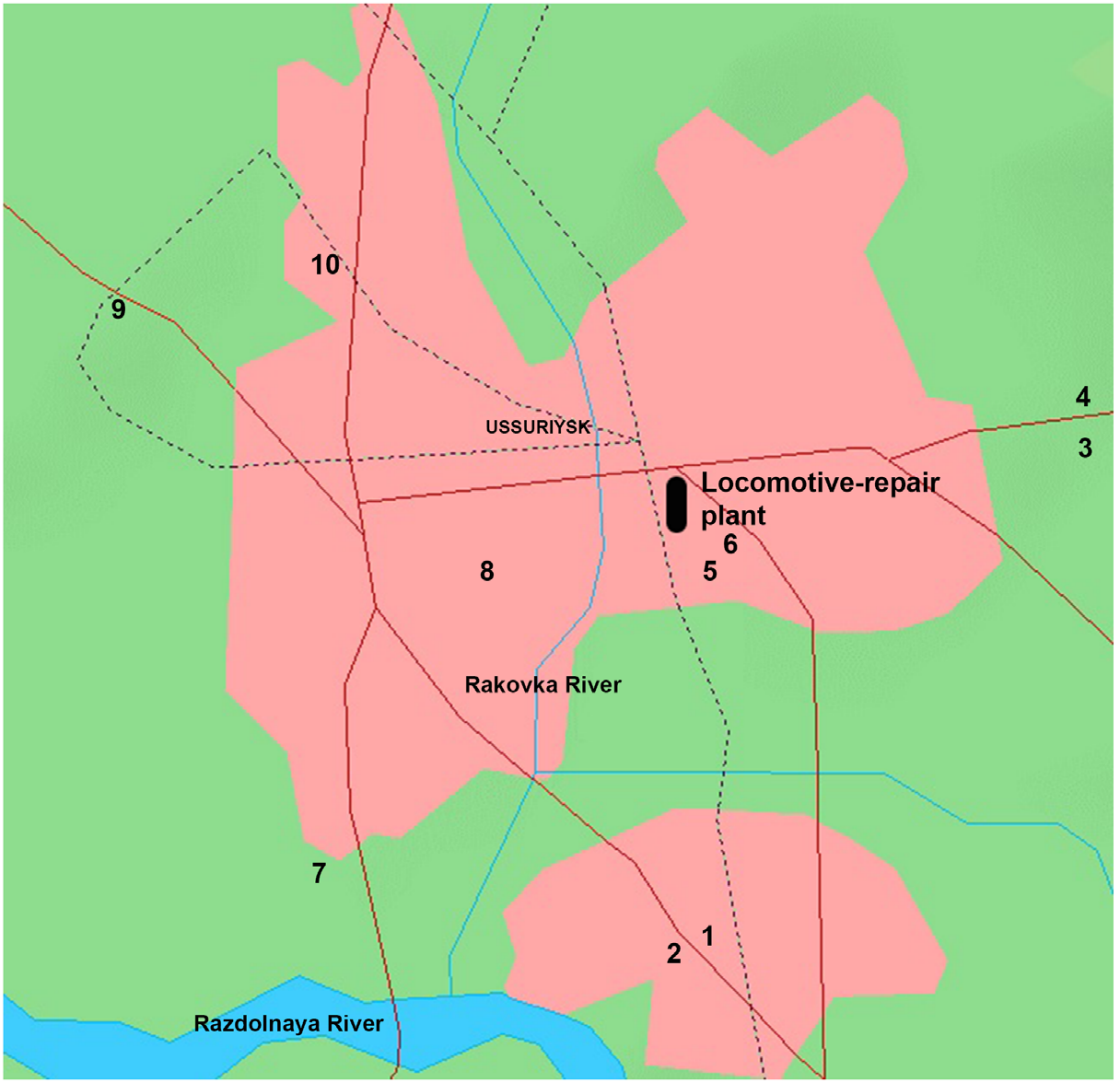
Schematic map of the locations of the sampling of snow in the territory of Ussuriisk (stations of sampling are described in Table 1. The black rectangle in the map denotes the locomotive-repair plant. (Earth Science and Remote Sensing Unit, NASA-Johnson Space Center. “The Gateway to Astronaut Photography of Earth.” <http://eol.jsc.nasa.gov/Info/use.htm>09/16/2014 14∶13∶21).

**Figure 2:**
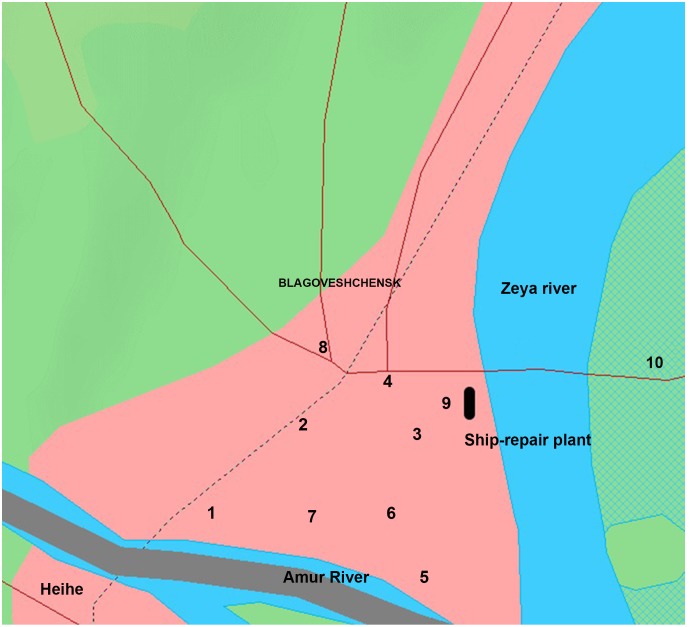
Schematic map of the locations of the sampling of snow in the territory of Blagoveshchensk (the stations of sampling are described in Table 2). The black rectangle in the map designates the ship-repair plant. (Earth Science and Remote Sensing Unit, NASA-Johnson Space Center. “The Gateway to Astronaut Photography of Earth.” <http://eol.jsc.nasa.gov/Info/use.htm>09/16/2014 14∶13∶21).

Snow sediments were collected for analysis of Me contaminants within several industrial areas of Blagoveshchensk and Ussuriisk (Russian Federation). The plans of cities were taken from OpenStreetMap (http://www.openstreetmap.org/copyright). The samples were gathered near large highways, repair plants, heat power plants and galvanic shops after snowfalls between 2010–2012 (Fig. 1–2 and Tables 1–2). Samples were collected from public areas; therefore, no specific permissions were required.

**Table 1:** Sampling areas in Ussuriysk.

Station	Sample station location
1	District “Sugar Factory.” Major road.
2	District “Sugar Factory.” Major road. Large company - JSC “Primorsky sugar.”
3	Bypass route on the leeward side. Road and park zone.
4	Bypass route - the opposite site. Road and park zone.
5	Industrial zone. Near Ussuri locomotive-repair plant (Blucher Avenue, 19), near railway station “Ussurijsk” 5 m from major road in public zone.
6	Industrial zone. The sample is taken by the house Blucher Avenue, 36, in front of the Ussuri locomotive-repair plant.
7	Utesnoe Village, park zone.
8	Down Town of the Ussuriysk with traffic 1500 car per hour and small boilers.
9	Large boiler, regularly on railroad tracks with carbon come.
10	Northern district of the city. There are several small boilers, and substantial flow of goods.

**Table 2:** Sampling areas in Blagoveshchensk.

Stations	Description of the sampling sites
1	First City Hospital. Parks.
2	Thermal power station, a coal-fired plant.
3	Exhibition center and highway.
4	Railway station.
5	Lenin Square. Shore of the Amur River. Major road.
6	Vicinity of shopping center “Mega.” Large motor isolation.
7	Crossroad of Gorky and Kalinin streets.
8	Kalinin street. A major ring road.
9	Theatralnaya street. A major ring road. Ship-repair plant (Pushkin street, 189).
10	Traffic police post on the outskirts of the city. Major road.

### Sample preparations

Snow sediments were chosen as sorbents for the assessment of airborne PM [20]. This method was employed to measure the level of Me NP in water samples derived from thawed snow sediments gathered from park zones (control) and the industrial sites. The snow samples were gathered from a 1 m^2^ area within 200–500 m around the galvanic shop. The top layer of freshly fallen snow was collected into 3-liter plastic polyvinylchloride containers. The snow samples were thawed, evaporated to 60 ml and sterilized. All samples collected from the clean areas (control, n = 3) and industrial zones (exposed, n = 3) were stored and kept in the dark at 4°C until processed. Two independent experiments were done to assess Me NP content in snow sediment collected from two cities (see Materials S1 for the details).

### Particle size distribution

For measurements of particle size distribution, a laser particle size analyzer supplied with Fritch MaS software (Analysette 22 NanoTec Fritsch, Idar-Oberstein, Germany) was employed. This technology can analyze PM size distribution of the wet or dry dispersion units separately or simultaneously with automated switching features. Optimal dispersion was accomplished in the NanoTec integral wet dispersion unit by using a combination of a robust, variable speed centrifugal pump with powerful ultrasonification, according to the manufacturer’s manual. The software controls all of the functions of the NanoTec wet dispersion unit. The sample was added in the open dispersion chamber on the top of the instrument. The samples (60 ml) were diluted in water (150 ml) and then tested.

### Scanning electron microscopy (SEM)

For scanning electron microscopy, the water from the samples was evaporated and the dry PM was covered with platinum using a turbo-pumped sputter coater Q150T (Quorum Technologies, Lewes, United Kingdom). The collected PM were characterized by X-ray diffraction (XRD) analysis. Morphology of PM was evaluated by SEM using Zeiss EVO 40XVP (Zeiss, Oberkochen, Germany) with an energy dispersive spectrometer INCA Energy (Oxford Instruments, Abingdon, United Kingdom) and Hitachi S-3400N (Hitachi, Tokio, Japan) with an energy dispersive spectrometer Ultra Dry (Thermo Fisher Scientific, Waltham, USA). Two microscopes were used to differentiate the size of collected Me NP.

### Measurements of Me in collected samples

A high resolution inductively coupled plasma mass spectrometer (HR-ICP-MS) employing Element XR (Thermo Fisher Scientific, Waltham, USA) was used for assessment of Me in collected snow PM samples. Prior to evaporation, the liquid portion of the samples was stored at 4°C and analyzed according to technique CV 3.18.05-2005 (standard by JSC “Water Research and Control Center;” http://aqua-analyt.com).

### Statistical Analysis

Results were compared by one-way ANOVA using the all pairwise multiple comparison procedures (Holm-Sidak method) or Dunnett’s multiple comparisons to control, and Student’s unpaired t test with Welch’s correction for unequal variances. All results are presented as means ± SE. P values of <0.05 were considered to indicate statistical significance.

## Results

**Figure 3:**
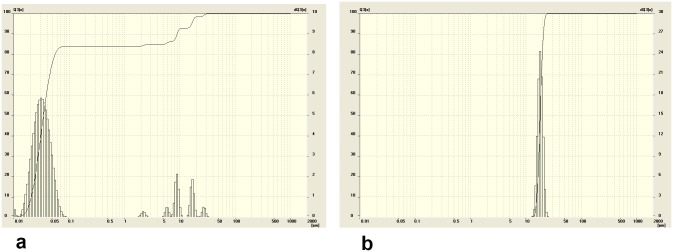
The profile of particle size distribution in snow samples collected from the western section**, Ussiriisk (point 6) (a).** The size of the particles from the samples collection ranges between 10–70 nm and between 2–3, 5–7, 7–10, 7–12, 20–30 and 30–40 µm. The profile of particle size distribution in snow samples collected from the bypass road, Ussuriisk (point 3) **(b)**. The size of the particles from the samples collection ranges between 14–23 µm.

The snow sediments were collected from Blagoveshchensk and Ussuriisk during 2010–2012. Snow samples were collected from the area of 200–500 m around galvanic shops, large highways and heat power stations. The maps of the areas and collection spots are presented in Fig. 1/Table 1, and Fig. 2/Table 2. Me NP were only found in snow sediments collected from areas in both cities near enterprises employing galvanic fabrication. In Ussuriisk, for example, Me NP were present in snow nearby the locomotive-repair plant and the carriage refrigerator depot (Fig. 3a, point 6). No Me NP were detected in control samples collected from clean/green region of a bypass road at the western part of the city (Fig. 3b, point 3) or other areas of Ussuriisk (Table 3, 4).

**Table 3:** Morphometric parameters of the particles in thawed snow samples collected from points 3 and 6 in Ussuriisk.

	point 6						point 3
Size, µm	0,01–0,07	2–3	5–7	7–12	20–30	30–40	14–23
Proportion, %	84	1	3	5	5	2	100
Arithmetic mean diameter, µm	2.06±0.12						18.26±1.21
Specific surface area, m^2^/sm^3^	171.15						0.13

**Table 4:** Morphometric data of the particles in thawed snow samples collected from points 1–5 and 7–10 in Ussuriisk.

Points	1	2	4	5	7	8	9	10
Arithmetic mean diameter, µm	519.41±33.2	104.7±9.0	146.99±8.8	386.19±22.1	432.22±38.9	15.60±1.2	120.85±9.4	7.48±1.0
Specific surface area, sm^2^/sm^3^	5554.4	3324.8	8331.6	2346.7	1436.9	5286.4	3264.4	9303.1

**Figure 4:**
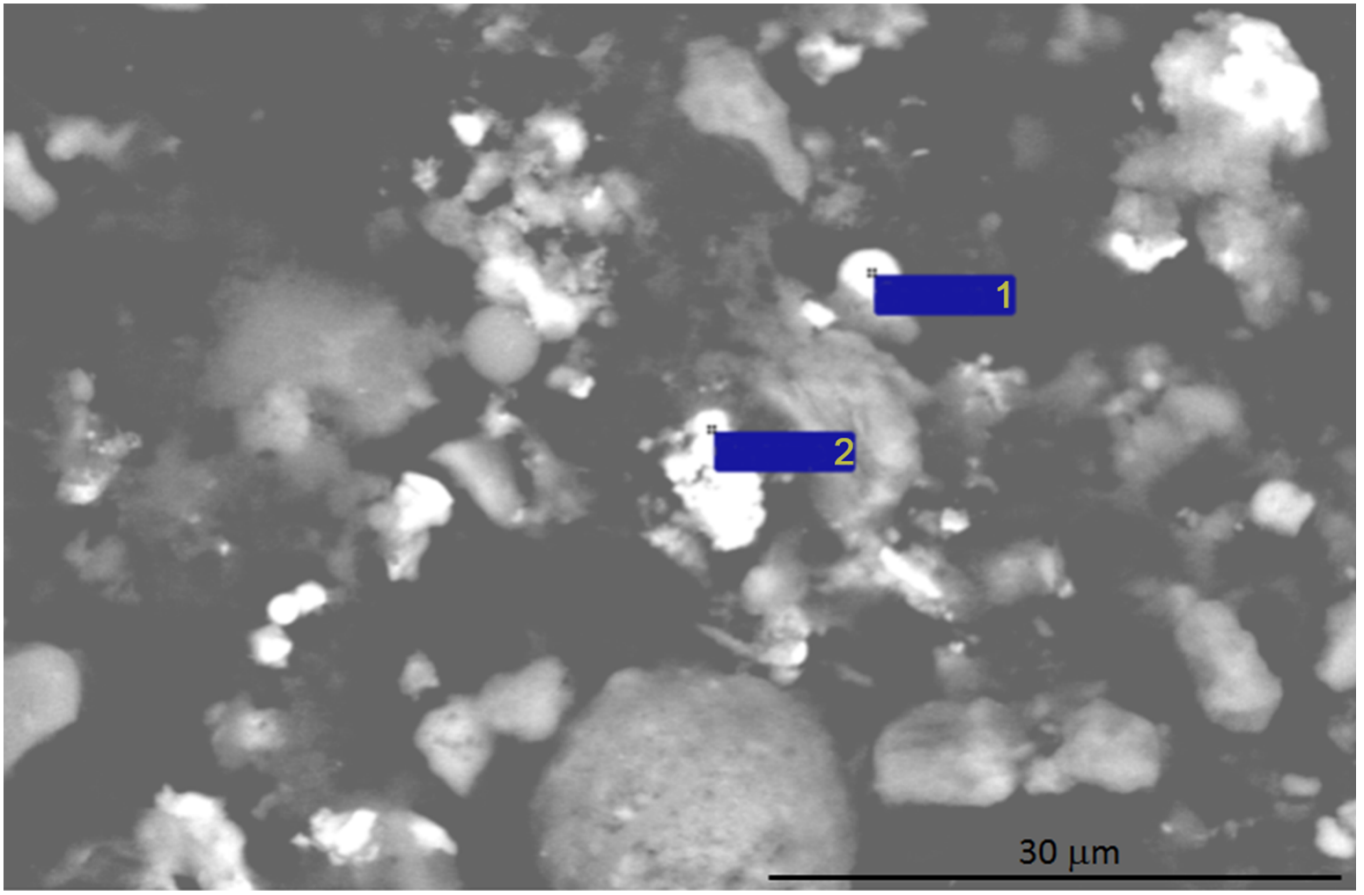
Images of the survey micrograph of particles from a snow sample in the western section of Ussuriisk (point 6), executed in reflection mode. White - metal particles. 1 and 2 indicating particles tested by EDAX method.

Apparently, an average particle size distribution of Me NP collected from clean areas fluctuates from 7.48±0.97 to 519.41±33.21 µm (Fig. 3b, Tables 3–4) while size distribution of Me NP gathered from industrial settings ranged 2.06±0.12 µm (Fig. 4, Tables 3).

The data showing surface area of Me NP collected from all areas in Ussuriisk are presented in Table 3–4. The Me NP collected from industrial point 6 (Table 3) had a surface area 1400-fold higher compared to those collected from clean area sources.

To confirm that these NPs contained metals, scanning electron microscopy and the energy dispersive X-ray spectral analysis (EDX-analysis) were used for assessment of a dry suspension of the collected snow sediments. The most characteristic images of the particles are shown in Fig. 4. The data presented in Table 5 indicates the results of EDX-analysis of two heavier particles shown in white in Fig. 4.

**Table 5:** Elemental composition of two Me microparticles tested in the snow samples collected from Ussuriisk (point 6) by EDAX method.

Element	Spectrum 1		Spectrum 2	
	mass. %	atom. %	mass. %	atom. %
O	7.11	20.52	24.20	50.44
Al			2.93	3.62
Si	3.18	5.24	4.12	4.89
Fe	89.71	74.24	68.75	41.05

All studied particles were smaller or comparable in size to the diameter of the electron beam focus of the SEM. Therefore, Si and Al were also detected by EDX-analysis from nearby aluminosilicate impurities (Table 5). Chemical analysis of the data presented in Table 5 revealed that iron in these NPs was in oxidized form. The shapeless (xenomorphic) particles were oxidized more strongly (Fe_2_O_3_) (range 2) than the spheroidal smaller particles (FeO) (range 1) (Fig. 4).

Quantitative analysis by HR-ICP-MS of Me composition of the collected PM in snow samples from ten different Ussuriisk city areas is presented in Table 6. The content of metals present in snow from Ussuriisk did not differ from each other by more than ten times and did not exceed toxic levels. The elevated level of Fe and Cr were detected in the samples gathered from points 5 and 6. These Me were present in oxidized and salt form. We propose that the mechanism of NPs formed in the atmosphere was due to high temperature and the etching and fast evaporation of galvanic solutions. Formed galvanic steam satiated by salts stayed airborne and then precipitated in metallic and/or oxidized forms. Our observation demonstrates that at a distance greater than 250 m, no metals NPs were found. Therefore, airborne NP found in the snow sediments gathered within 200 to 250 m from galvanic shops were enriched with emitted Me. Additionally, the paradigm of wind flow mapping strictly corresponds to the NPs transferred from the plants.

**Table 6:** Results of the mass spectrometry of samples of the snow collected from 1–10 districts in Ussuriisk (the data presented in ppb).

district/element	^111^Cd	^207^Pb	^27^Al	^52^Cr	^56^Fe	^59^Ni	^63^Cu	^66^Zn
1	-	-	2.47±1.15	-	0.91±0.32	0.77±0.04*	1.32±0.25	4.73±1.02*
2	-	-	0.63±0.05*	-	0.29±0.03*	3.61±0.27*	2.74±0.76*	4.32±0.41*
3	0.13±0.02	0.02±0.004	3.46±1.04	-	1.22±0.11	1.46±0.09	0.77±0.8	12.2±1.54
4	0.03±0.01*	0.02±0.004	6.77±1.02*	0.09±0.01*	1.71±0.26	1.57±0.24	0.82±0.12	6.97±1.04
5	1.3±0.03*	5.52±0.36*	9.2±1.29*	2.71±0.18*	22.10±2.19*	4.71±0.62*	1.72±0.03	31±4.59*
6	0.6±0.04*	8.75±0.88*	29.1±3.79*	3.02±0.21*	120.02±13.89*	6.6±0.91*	2.33±0.12*	223±31.45*
7	0.03±0.01*	0.77±0.12	3.62±0.54	0.09±0.04	9.66±1.45*	0.35±0.05*	0.61±0.09	276±41.34*
8	0.07±0.01	1.57±0.23	3.95±0.59	0.25±0.04*	4.12±0.62*	0.39±0.06*	0.81±0.12	234±35.14
9	0.05±0.01	0.26±0.04*	6.56±0.98*	0.32±0.05*	3.54±0.53*	1.52±0.23	3.93±0.59*	3.39±0.51*
10	0.03±0.01*	0.82±0.12*	7.29±1.09*	0.1±0.01	8.36±1.25*	0.39±0.06*	0.41±0.06	273±41.01*

“-” below detection limit, *p<0.05, vs control (point 3).

**Figure 5:**
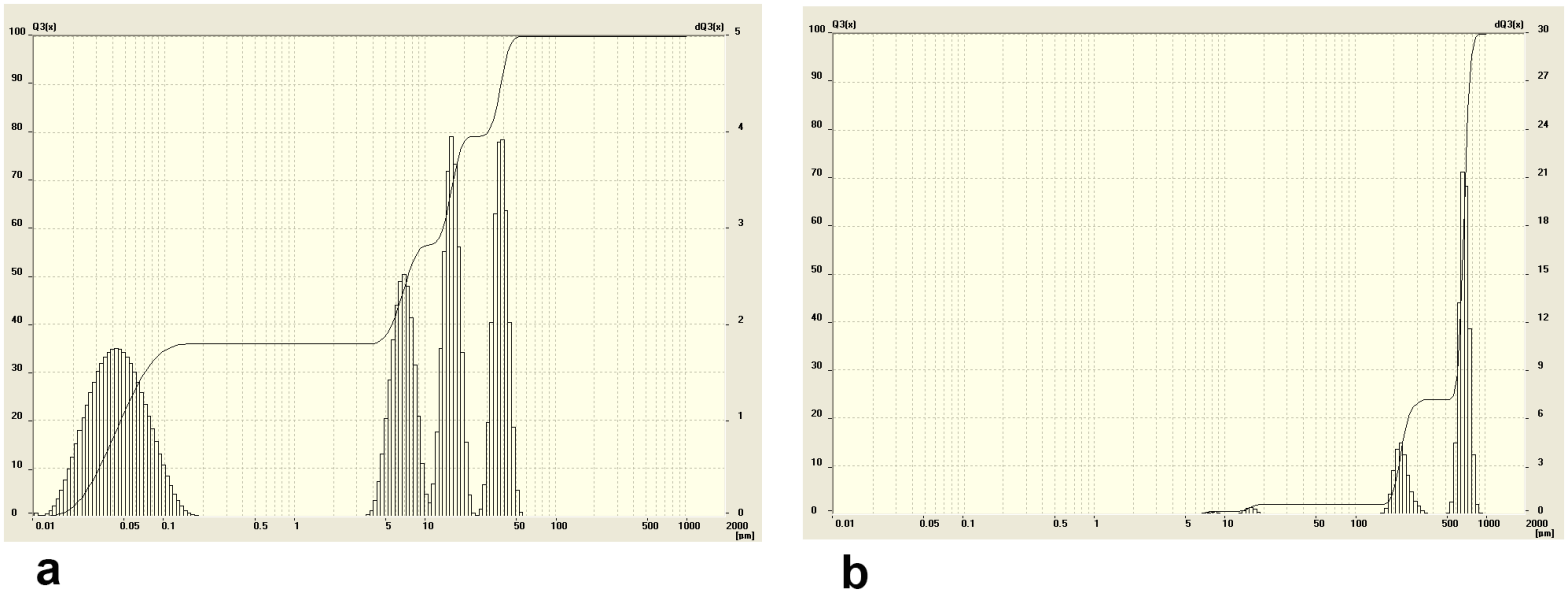
The profile of particle size distribution in snow samples collected from the Theatralnaya street, Blagoveshchensk (point 9) (a). The size of the particles from the samples collection ranges between 10–120 nm, 4–10, 10–30 and 40–50 µm. The profile of particle size distribution in snow samples collected from the First City Hospital, Blagoveshchensk (point 1) **(b)**. The size of the particles from the samples collection ranges between 7–9 µm, 13–19, 170–350 and 500–900.

In Blagoveshchensk’s industrial areas, Me NP were present within a ship-repair plant (Fig. 5a, Table 7, point 9, Theatralnaya st.).

**Table 7:** Morphometric parameters of PM derived from snow samples collected at points 1–10 in Blagoveshchensk.

	point 9				point 1			
Size, µm	0.01–0.12	4–10	10–30	40–50	7–9	13–19	170–350	500–900
Proportion, %	36	20	24	20	1	3	23	73
Arithmetic meandiameter, µm	13.07±1.11				579.12±36.24			
Specific surfacearea, m^2^/sm^3^	56.41				0.022			

In contrast, no Me NP were detected in the samples gathered from First City Hospital park at point 1 (Figure 5b and Table 7).

Interestingly, the differences in average diameter of nanoparticles in dirty zones was 44 times less compared to clean ones, showing that nano-sized particles are predominately found within cites where galvanic plants are located. It also has to be mentioned that the specific surface area of the particles collected in Blagoveshchensks were 256 fold higher in dirty areas compared to clean areas (Figure 5a and 5b).

The average arithmetic diameter of particles collected from other points (2–8, 10) in Blagoveshchensk fluctuated ranging from 8.47±0.61 to 288.83±12.36 µm (Table 8).

**Table 8:** Morphometric parameters of PM in collected snow samples (Blagoveshchensk) (points 2–8, 10).

Points	2	3	4	5	6	7	6	8	10
Arithmetic mean diameter	8.5±0,6	39.7±2.4	586.7±21.2	53.6±4.9	288.8±12.4	12.2±1.0	105.6±7.5	750.2±46.5	14.8±1.8
Specific surface area, sm^2^/sm^3^	7139.7	1532.1	686.4	3051.9	730.0	4956.0	587.5	582.8	4066.0

**Figure 6:**
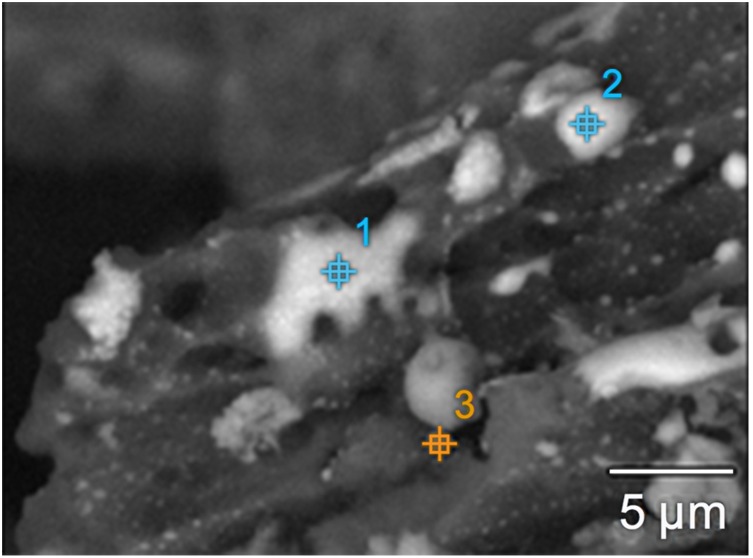
Image of the survey micrograph of particles from a snow sample around a ship-repair plant in Blagoveshchensk (point 9) imaged in reflection mode. White - metal particles. The large microsized “drops” consist of nanodimensional particles. Numbers 1–3 indicating particles tested by EDAX method.

The chemical characterization of the particles seen in Figure 6 (marked as 1, 2 and 3) is presented in Table 9.

**Table 9:** Elemental composition of three Fe microparticles tested in the snow samples collected from Blagoveshchensk (point 9) by EDAX method.

Element	Spectrum 1	Spectrum 2	Spectrum 3
	Macc. %	Macc. %	Macc. %
C	8.52±0.18	8.54±0.16	26.72±0.32
O	40.18±0.61	37.58±0.57	18.30±0.46
Na			1.03±0.07
Al	8.49±0.18	8.79±0.16	7.21±0.16
Mg	1.85±0.15	1.64±0.14	0.82±0.07
Si	1.06±0.13	0.96±0.07	5.73±0.14
K			0.52±0.07
Cl			0.56±0.10
Ca	15.15±0.29	17.05±0.29	12.83±0.27
Mn		1.63±0.21	1.54±0.22
Fe	24.76±0.80	23.81±0.75	24.73±0.80
Sum	100.00	100.00	100.00

**Figure 7:**
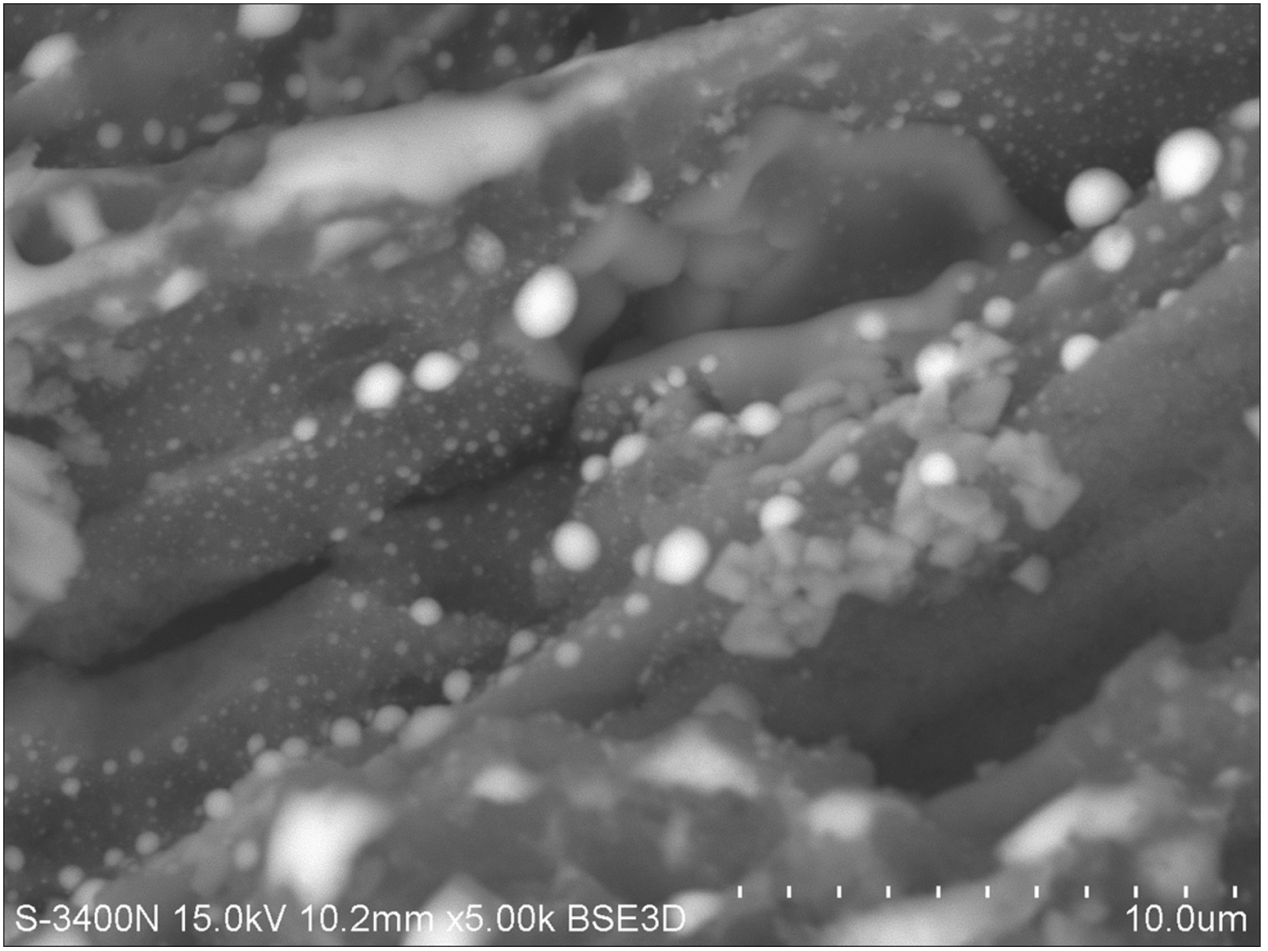
Image of the survey micrograph of particles derived from a snow sample collected from the area of a ship-repair plant in Blagoveshchensk (point 9). White - metal particles. NPs are observed to be absorbed by organic detrite (identity by morphology and C-peak in EDAX). Magnification: 5000×. Images are in reflection mode.

The large ferriferous particles detected by SEM are shown in Figure 4, 6 and 7. The surface of these PM was covered with nano grains, most likely formed due to aggregation of small iron particles (Figure 7).

The measurements by HR-ICP-MS indicate the low metallic concentrations of Fe and Cr in the samples from Blagoveshchensk (Table 10). Different Me composition was observed in the particles collected from ten different city areas. The content of metals collected from Blagoveshchensk did not differ from each other by more than ten times and did not exceed toxic levels. NP gathered from Ussuriisk areas had fewer metals than NP collected from Blagoveshchensk. This may be attributed to less numbers of airborne NP in areas of reduced traffic.

**Table 10:** Metal composition in snow samples collected from 1–10 districts in Blagoveshchensk (Mass spectrometry, the data presented in ppb).

district/element	^111^Cd	^207^Pb	^27^Al	^52^Cr	^56^Fe	^59^Ni	^63^Cu	^66^Zn
1	0.03±0.01	0.02±0.04	6.77±1.02	0.09±0.01	1.71±0.26	1.57±0.24	0.82±0.12	6.97±1.04
2	0.21±0.01*	-	1.92±0.3*	0.12±0.01	-	2.2±0.11*	0.85±0.09	2.89±0.83*
3	0.14±0.02*	1.27±0.09*	4.34±0.87	0.91±0.04*	2.12±0.12	1.71±0.16	0.29±0.05*	6.78±0.93
4	0.49±0.03	1.42±0.07*	0.14±0.02*	-	1.42±0.11	2.42±0.32*	-	-
5	0.33±0.02*	0.29±0.02	4.66±0.92	-	1.13±0.09*	1.72±0.12	0.92±0.09	2.72±0.34*
6	-	1.11±0.08*	0.92±0.11*	0.27±0.03*	3.23±0.46*	0.88±0.08*	1.23±0.08*	-
7	0.39±0.01*	1.12±0.06	0.72±0.03*	1.09±0.03	0.93±0.08*	1.71±0.11	1.21±0.11	1.76±0.12*
8	0.52±0.05*	0.22±0.03*	2.72±0.94*	-	2.23±0.14	-	1.52±0.34*	0.57±0.02*
9	0.2±0.03*	2.56±0.38*	23.2±3.47*	0.25±0.04*	6.12±0.11*	1.12±0.17*	0.38±0.06*	310.8±46.45*
10	0.56±0.08*	-	2,64±0.33*	-	2.06±0.11	0.96±0.7*	-	2.69±0.31*

“-” – below the detection level; *p<0.05, vs control (point 1).

## Discussion

A number of epidemiological studies have reported a strong correlation between the level of particulate air pollution and increased morbidity and mortality rates in both adults and children [21]–[24]. Particle count, composition, and surface properties are recognized as important for dosimetry and the assessment of adverse outcomes caused by air pollution [25]. The deposition of particles in the respiratory tract depends predominantly on their size; larger particles will accumulate in the upper and larger airways, while smaller particles will penetrate into the alveolar spaces. Thus 90% of inhaled 1 µm particles are deposited in the nasopharyngeal region while 20 nm particles have the highest deposition efficiency in the alveolar region (∼50%) [26].

Me nanoparticles are very reactive and are known to cause wide range of toxic responses [27]–[29]. In welders exposed to a variety of fumes and gases (containing a mixture of oxides and salts of metals), the rate of pulmonary morbidity was relatively high [30]–[33]. An early meta-analysis among shipyard, mild steel and stainless steel welders revealed an increased lung cancer risk among all types of workers [34].

We have found elevated numbers of hard and soluble Me NP in snow sediments collected in close vicinities of galvanic manufacturers in two Russian cities. We are the first to report that hard and soluble Me NP consisting Pb, Al, Cr, Fe, Ni, Cu and Zn were detected around galvanic shop settings. Using different analytic techniques we observed that the size distribution of tested Me NP was within the 10–120 nm range.

Potential exposure to Fe NP and Cr NP during production is an area of concern. Fe NP have unique magnetic properties which gives a high potential use in several biomedical applications, including magnetic drug targeting, magnetic detection, hyperthermia and magnetic resonance imaging [31]. Chromium is also an important industrial metal used in various processes, sometimes in the nanosized form.

A number of regulations have been implemented by the Department of Labor of Russian Federation targeting the safety of galvanic manufacture workers. There are two major regulatory documents “Sanitary standards designed for the industrial enterprises” and “The instruction on labor protection for the galvanizer man” which were designed to regulate the use of protective measures in the galvanic industry (CH 245-71, 1972; TOI R-31-205-97, 2011). In particular, the “Sanitary Standards Designed for the Industrial Enterprises” emphasizes the need to control exposure level around electrolytic plating workstations. Depending on the chemical galvanic technology used, certain areas were assigned for assessment of hazard identification providing industrial hygiene control in the area ranging from 100 m to 500 m. For good manufacturing practice, proper industrial control and a safe work environment, testing of airborne toxic fumes, Me and gases are recommended to be performed every six months (CH 245-71, 1972; TOI R-31-205-97, 2011). Additionally, when galvanic and plating technology is changed, all tests of airborne emitted galvanic contaminants are advised to be re-assessed. Furthermore, to capture harmful emissions, the use of a ventilation exhaust system has been strongly recommended to provide proper work safety (“The instruction on labor protection for the galvanizer man” TOI R-31-205-97, 2011) [35]. Unfortunately, the industrial ventilation devices currently used are not supplied with filters that provide efficient capturing of airborne nano-sized PM and fumes emitted during galvanic manufacturing.

## Conclusions

Me nanoparticles of Fe, Cr and Fe/Cr-alloy are potentially released into the urban air during galvanic production. No Me NP were detected in snow sediments collected from clean areas in two industrial cities. Elevated levels of Me were detected in snow sediments in close proximity to galvanic shops. Future epidemiological studies are needed to determine whether the airborne Me NP that were found elicit adverse health effects.

## Supporting Information

Materials S1 (DOC)
