# Supplementary material for: Galvanic Manufacturing in the Cities of Russia: Potential Source of Ambient Nanoparticles
Source: PLoS One. 2014 Oct 20;9(10):e110573. doi: 10.1371/journal.pone.0110573 (PMC4203814; doi:10.1371/journal.pone.0110573)
Supplement: Materials S1 — (DOC) [file pone.0110573.s001.doc]

**Materials S1:**

***Area of samples collection.*** All of the samples were collected in Blagoveshchensk (10 pairs) and in Ussuriisk (10 pairs).

All of the samples were collected in the winter of 2010-2012 in different city areas during snowfalls. The samples were collected in areas with different environmental pressures and were also collected near the primary sources of dusting - large highways and heat power main lines. Points were chosen, considering the average month wind rose (Feb 2010-2012), at a distance of about 1 km from each other round the enterprise with galvanic shops.

Blagoveshchensk is the administrative center of the Amur region (Far East, Russian Federation), being located in the southwest of Zeya-Bureya Plain, on the left coast of Amur River and at a confluence of the Zeya River (Fig. 1 and Table 1).

The city relief is generally flat with small hills in the suburbs. In the city, which has a population of 220,000 people (2011); there are several large sources of dust: power plants, over 10 boiler rooms and approximately 90,000 cars (for 2011) according to the Department of Traffic Police of the Amur region (http://28.gibdd.ru). In Blagoveshchensk, the climate is sharply continental with monsoonal periods reflected in significant annual and daily fluctuations of air temperatures and a prevalence of summer precipitation. Summer is hot but short and rainy with a significant amount of sunshine. Winter is cold and dry with a light snow cover. During the days of selection (2010-2012) of the tests in Blagoveshchensk, the wind was blowing predominantly in the southern and southeastern directions.

Ussuriisk is the second largest city in the Primorsky Region, being situated 112 km to the north from Vladivostok along the Razdol'naya River at a confluence of the Rakovka and Komarovka Rivers (Fig. 2 and tab. 2).

Over 160,000 people (2012) live in the city. A large fraction of the industrial enterprises in the city use outdated equipment, there is a lack of wastewater treatment plants in the city and a considerable quantity of emissions in the environment includes various technogenic wastes. The primary sources of air pollution in the territory of Ussuriisk are city and public transportation, the heat power complex and industrial enterprises. Winter is moderate with a light snow cover. The wind blew primarily in the northern direction during the days of sampling (2010-2012) in Ussuriisk.
